# Low Doses of Resveratrol Protect Human Granulosa Cells from Induced-Oxidative Stress

**DOI:** 10.3390/antiox10040561

**Published:** 2021-04-04

**Authors:** Beatriz Moreira-Pinto, Lia Costa, Eduarda Felgueira, Bruno M. Fonseca, Irene Rebelo

**Affiliations:** 1 UCIBIO, REQUIMTE, Laboratory of Biochemistry, Faculty of Pharmacy, University of Porto, Rua de Jorge Viterbo Ferreira no. 228, 4050-313 Porto, Portugal; abeatriz_pinto@hotmail.com (B.M.-P.); irebelo@ff.up.pt (I.R.); 2 Unidade de Medicina da Reprodução Dra, Ingeborg Chaves-Centro Hospitalar de Vila Nova de Gaia/Espinho, R. Dr. Francisco Sá Carneiro, 4400-129 Vila Nova de Gaia, Portugal; liacosta.bio@live.com (L.C.); eduardafelgueira@chvng.min-saude.pt (E.F.)

**Keywords:** resveratrol, antioxidants, dietary supplements, fertility, granulosa cells

## Abstract

Resveratrol is a phytoalexin present in plant-derived foods, including grape’s skin, cocoa, and peanuts. Evidence suggests that it has beneficial effects on human health because of its antioxidant properties. However, there is limited knowledge about the part played by resveratrol in ovarian function. In this paper, the influence of resveratrol on granulosa cells (GC) was evaluated. In addition to being the main estradiol producers, GC are in direct contact with the oocyte, playing a fundamental role in its growth and development. The cell line COV434 and human granulosa cells (hGC), obtained from women undergoing assisted reproductive technology (ART), were used. GC were treated with resveratrol (0.001–20 μM) at different times (24–72 h). Low concentrations of this compound suggest a protective role, as they tend to reduce ROS/RNS formation after inducement of stress. On the contrary, high concentrations of resveratrol affect GC viability and steroidogenic function. As it may act as a direct modulator of GC oxidative balance, this work may help to clarify the impact of resveratrol on GC and the usefulness of this antioxidant as adjunct to infertility treatments.

## 1. Introduction

Resveratrol (3,4’,5-trihydroxystilbene, RES) is a plant-derived polyphenol stilbene synthesized in response to mechanical injury, ionizing radiation, and fungal attacks [1]. RES is present in several berries, grapes’ skin (especially red grapes), cocoa, peanuts, and other plant-derived foods [2]. Its potential health benefits have been associated with the phenomenon called “French Paradox”: Despite the high intake of dietary saturated fat in France, the percentage of cardiovascular disease is relatively low in this country and this may be attributable in part to high wine consumption containing RES [3]. In recent years, RES consumption as a dietary supplement is wide spreading regardless of its low bioavailability as a result of rapid and extensive metabolism in the liver and intestine [4]. The growing scientific interest for RES is due to its antioxidant and anticarcinogenic activity, which has been described in numerous publications [5,6]. Studies suggest that this important antioxidant compound can help prevent a wide range of age-related diseases, including cardiovascular disease, diabetes, cancer, and neurodegeneration, among others [7]. They also agree with the efficacy of RES in mitochondrial regulation by targeting various molecular pathways [8]. In addition, RES is considered a phytoestrogen, based on its ability to bind to estrogen receptors to enhance endothelial function in breast cancer cell lines [9,10]. However, little is known about the role of RES in vital biological functions such as reproduction and ovarian function.

Extensive clinical investigations indicate that dietary and lifestyle preferences can be associated with follicle growth and ovulation rates in women undergoing assisted reproduction technology (ART) [11,12]. Several patients suffer from infertility with no cause clearly identified, suggesting that emotional stress, environmental factors, and nutritional status may play a role on their reproductive function [13,14]. In particular, vitamin D at high levels is associated with an increased likelihood of a successful pregnancy, and may be especially beneficial for patients with polycystic ovary syndrome (PCOS) in reducing hyperandrogenism [15]. Similarly, myo-inositol helps in reducing excess androgen and insulin resistance [16]. Therefore, antioxidant supplementation may contribute to overcoming complications such as immature oocyte and oxidative stress in the human ART context [17]. Oxidative stress (OS) occurs when there is an excessive production of Reactive Oxygen Species (ROS) or when the antioxidant defense mechanisms are weakened [18]. OS plays an important role both in cases of pathology-associated infertility and in cases of idiopathic infertility [19]. Since it is known that a sufficient intake of antioxidants can decrease the risk of ovulatory infertility, women with fertility complications tend to self-medicate with antioxidant supplements [20,21].

Granulosa cells (GC) play a major role in ovarian follicle and oocyte growth and maturation due to two essential functions: Cell proliferation and steroidogenesis [22]. Thus, GC are important to evaluate and predict follicular health [23]. In addition, these cells can be disrupted by environmental factors such pollutants or diets [24,25]. For these reasons, it is of great interest to understand the RES effects on human granulosa cells. Therefore, the core of this study was to evaluate the direct effects of RES on GC viability, steroidogenic function, and oxidative stress in vitro using both GC from women undergoing fertility treatments and the immortalized human GC line (COV434) for a better comprehension of its mechanisms of action.

## 2. Materials and Methods

### 2.1. Chemicals

Dulbecco’s modified eagle medium/F12 (DMEM/F12), resveratrol (R5010), Höechst 33342, methylthiazolyldiphenyl-tetrazolium bromide (MTT), dimethyl sulfoxide (DMSO), carbonyl cyanide m-chlorophenylhydrazone (CCCP), tert-butyl hydroperoxide (TBHP), and dichlorodihydrofluorescein diacetate (DCFH-DA) were bought from Sigma–Aldrich Co., St. Louis, MO, USA. Antibiotic-antimycotic (AB-AM) was from Grisp. Trypsin (2.5%) and 3,3′- dihexyloxacarbocyanine iodide (DiOC6) was from Gibco/Invitrogen Corporation, Carlsbad, CA, USA. Fetal bovine serum (FBS) came from Biochlome. The Pierce Lactate Dehydrogenase (LDH) cytotoxicity assay kit was from Thermo Fisher, Life Technologies. Dibutylphthalate Polystyrene Xylene (DPX) was from VWR-Prolabo. Percoll came from GE Healthcare, Buckinghamshire, UK). The adenosine triphosphate (ATP) assay kit (ab83355) was from Abcam, Cambridge, UK.

### 2.2. Methods

#### 2.2.1. Study Design

Follicular fluid (FF) samples containing GC obtained from women undergoing assisted reproductive technology (ART) were collected, with their informed consent, at Unidade de Medicina da Reprodução Dra. Ingeborg Chaves-Centro Hospitalar de Vila Nova de Gaia/Espinho. A total of 34 patients were involved in this study with a mean of age of 34 years old. The inclusion criteria were women with tubal factor infertility and couples with male factor-associated fertility undergoing ART. Women with endometriosis, tumor, or ovarian cysts, as well as women with hormonal factor-associated infertility were excluded. RES supplementation was not prescribed to the patients who took part in this study. All the procedures were carried out in conformity with the Declaration of Helsinki, endorsed by the local ethical committee and approved by Comissão de Proteção de Dados (Proc. no.764/2017).

#### 2.2.2. Collection and Isolation of Primary Human Granulosa Cells (hGC)

Ovarian stimulation was performed accordingly to medical evaluation. When the follicles reached the adequate number and size, final maturation was induced, and oocyte retrieval was performed. For these reasons, a higher variability, acquired from the donors and ART procedures, may have occurred on these cells. Throughout oocyte aspiration FF was collected, the oocytes were isolated, and then removed for ART. The remaining FF was transferred to 50-mL polypropylene tubes. The GC were isolated and purified in accordance with a previously published protocol by Sluss et al. and individually cultured [26]. In summary, FF samples were centrifuged at 300× *g*, at 4 °C for 10 min, and the pellet was resuspended in DMEM/F12 medium and added to a Percoll:PBS density gradient in a ratio 1:1. Then, the tubes were centrifugated at 900× *g* for 15 min at 4 °C. hGC were obtained at the interface of the FF and Percoll, washed, and resuspended in cell culture medium.

#### 2.2.3. Cell Culture

Since a human granulosa cell line is a more robust and homogeneous model, the human granulosa cell line (COV434), which is derived from a primary ovarian solid tumor [27], was also used. Both hGC and COV434 cellular models remained under the same conditions. For all experiments, cells were seeded in DMEM/F12 medium supplemented with 1% AB-AM and 5% FBS. After 24 h, they were treated with different concentrations of RES in cell culture medium with 1% AB-AM and 2% FBS.

#### 2.2.4. Cell Viability Assays

COV434 and hGC were plated at a density of 5 × 10^4^ cell/well and 7.5 × 10^4^ cell/well, respectively, in 96-well plates (Sigma–Aldrich, CLS3340). After 24 h, the cells were treated with RES (0.001–20 μM) and incubated for another 24, 48, or 72 h, depending on the experiment. The concentrations were chosen based on the lowest RES concentration found in the circulating plasma of human studies on the absorption and bioavailability of this compound [28]. The higher concentrations were chosen in order to mimic supraphysiological conditions and study a broader range of effects that RES can have. In control experiments, cells were exposed to DMSO (RES vehicle) at concentrations similar to concentrations of DMSO at 20 μM of RES (˂0.01%). At these concentrations, DMSO did not affect cell viability. To determine cell viability, MTT at a final concentration of 0.5 mg/mL was added and incubated for another 3 h at 37 °C. A solution of DMSO:isopropanol (ratio 3:1) was used to dissolve the formed purple formazan crystals that were spectrophotometrically quantified at 540 nm after 15 min of shaking. The LDH released into the culture medium was assessed using the CytoTox 96 nonradioactive cytotoxicity assay kit as instructed by the manufacturer. The intensity of red formazan was quantified at 490 nm using BioTek Power Wave XS plate reader.

#### 2.2.5. Morphological Analysis

In order to evaluate morphological changes at the cellular level resulting from RES exposure, Giemsa staining was performed [21]. Moreover, to evaluate nuclear changes Höechst staining was performed [29]. Cells were cultured in 24-well plates (Sigma-Aldrich, St. Louis, MO, USA, SIAL0526) with coverslips at a density of 30 × 10^4^ cell/well for COV434 and 70 × 10^4^ cell/well for hGC. After 24 h, RES (1–5 μM) was added to the cells. Forty-eight hours later, GC were observed under a phase contrast microscope (Eclipse E400, Nikon, Japan) and prepared for Giemsa staining. Cells were fixed with methanol, stained with Giemsa staining solution for 30 min and observed under a bright field microscope equipped with image analysis software LeicaQWin. For Höechst staining, cells were fixed, subjected to 0.5 μg/mL Höechst 33342 for 30 min, and further examined under a fluorescence microscope (Eclipse CI, Nikon, Japan) fitted with an excitation filter with maximum transmission at 360/400 nm.

#### 2.2.6. Mitochondrial Membrane Potential (Δ*Ψ*_m_) Assessment, Mitochondrial Function (ATP assay), and Intracellular Reactive Oxygen and Nitrogen Species (ROS/RNS) Production

COV434 and primary GC were seeded in 96-well plates (BD Falcon, 353296; 353376) and treated with different concentrations of RES. After 24 h, for the mitochondrial membrane potential (ΔΨ_m_) study, RES was added (5–20 μM) and the cells incubated for another 24 or 48 h. The mitochondrial membrane-depolarizing agent CCCP (10 μM) was used as a positive control [30]. Cells were incubated with CCCP for 15 min before adding DiOC_6_ probe for 30 min at 37 °C [21]. Fluorescence was then measured in a Microplate Fluorimeter (BioTek Instruments, Winooski, VT, USA) (excitation: 488 nm; emission: 525 nm).

For the determination of ATP levels, GC were seeded and 24 h later the RES was added (0.001–10 μM). After 24, 48, or 72 h, we used the ATP colorimetric/fluorometric assay kit (#ab83355; Abcam) in agreement with the guidelines given by the manufacturer.

For the purpose of quantification of intracellular reactive oxygen and nitrogen species (ROS/RNS) generated, GC were seeded and 24 h later, RES (0.001–5 μM) was added. After 48 or 72 h, cells were incubated with H_2_O_2_ (200 μM) for 20 min, with H_2_O_2_ considered as a positive control for this experiment [31]. Then, the DCFH-DA probe was added and cells were protected from the light for 1 h at room temperature [31]. The Microplate Fluorometer was used to measure fluorescence that is proportional to cellular levels of ROS/RNS.

To explore long-term antioxidant potential, cells were treated with different concentrations of RES for 72 h and incubated for 1 h with the DCFH-DA probe, prior to treatment with TBHP (5 μM). The protocol was adapted from Kim et al. [32]. Finally, ROS levels were measured using the Microplate Fluorometer.

#### 2.2.7. DNA Isolation and Hormonal Quantification by ELFA

In order to study their steroidogenic function, COV434 and hGC were cultivated in 24-well plates (Sigma-Aldrich, St. Louis, MO, EUA. SIAL0526) at a density of 30 × 10^4^ cell/well and 70 × 10^4^ cell/well, respectively. On the next day, RES was added at a final concentration of 0.01 and 5 μM. In addition, 1 unit of follicle-stimulating hormone (FSH) and 4-androstenedione (Sigma-Aldrich, USA), dissolved in DMSO, were added. After 72 h of incubation, cell culture media were collected, centrifuged, and stored at –80 °C. VIDAS^®^ Estradiol II kits (bioMérieux SA, Marcy l’Etoile, France) were used to study the estradiol secretion by resorting to the enzyme-linked fluorescent assay (ELFA). DNA isolation was performed using TripleXtractor reagent, (GRiSP Research Solutions, Porto, Portugal), and quantified in the NanoDrop ND-1000 Spectrophotometer (NanoDrop Technologies,Inc., Wilmington, DE, USA). Hormone levels were normalized to cell DNA.

#### 2.2.8. Gene Expression Analysis by RT-PCR

To ascertain the amount of mtDNA relative to nDNA, RT-PCR was carried out by measuring the proportion of the mitochondrial 16S rRNA gene and the nuclear GAPDH gene. In line with this, GC were plated in 24-well plates (Sigma-Aldrich, SIAL0526) and different concentrations of RES (0.001–10 μM) were added. DNA was extracted with TripleXtractor reagent, (GRiSP Research Solutions, Porto, Portugal) and amplified with specific primers using the KAPA SYBR^®^ FAST qPCR Master Mix 2 × kit (Kapa Biosystems, Woburn, MA, USA) in the MiniOpticon Real-Time PCR Detection System (Bio-Rad Laboratories, Hercules, CA, USA), as per the kit’s protocol. Primer sequences and RT-PCR conditions for 16rRNA and GAPDH were as follows: 16S rRNA forward primer *ACTTTGCAAGGAGAGCCAAA* and reverse primer *TGGACAACCAGCTATCACCA;* annealing temperature (AT):59 °C; and GAPDH forward primer *GGATGATGTTCTGGAAGAGCC* and reverse primer *AACAGCCTCAAGATCATCAGC;* AT: 60 °C. Relative fold expression was analyzed by the 2^–ΔΔCt^ method.

#### 2.2.9. Statistical Analysis

Statistical analysis was carried out using the one- or two-way analysis of variance (ANOVA) test followed by the post-hoc Tuckey’s and Bonferroni test, respectively. In this line, one-way ANOVA was used for the quantification of ROS after stress induction, estradiol, and gene expression (16RNA/GAPDH) and two-way ANOVA for cell viability (MTT), LDH release, mitochondrial membrane potential, and ROS after 48 and 72 h. Means under comparison were obtained from at least three (maximum seven) independent experiments performed in triplicate. The results shown graphically are expressed as means ± SEM (standard error mean). A *p*-value < 0.05 was considered as statistically significant, although other *p*-values were also reported. Statistical analysis was conducted using GraphPad Prism software 7.0 (GraphPad Software, Inc., San Diego, CA, USA).

## 3. Results

### 3.1. RES Effects on GC Viability

**Figure 1:**
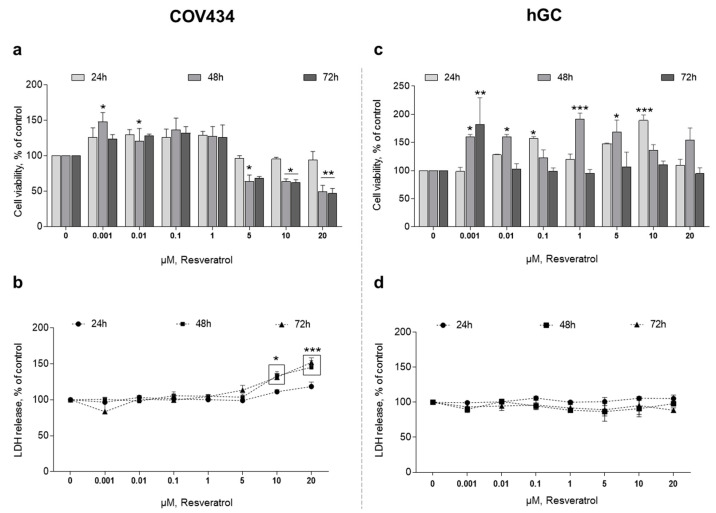
Resveratrol (RES) effect on COV434 and human granulosa cell (hGC) viability. MTT(**a**,**c**) and LDH (**b**,**d**) assays after treatment with different concentrations of RES (0.001–20 µM) at 24, 48, and 72 h. Results are related to the control and expressed as mean ± SEM of at least five independent experiments performed in triplicate. Significant differences between control and treated cells are indicated as * (*p* ˂ 0.05), ** (*p* ˂ 0.01) and **** (*p* ˂ 0.0001).

After 48 h of treatment, RES 0.001–0.01 µM promotes a significant increase in COV434 cell viability (Figure 1a). On the contrary, when we increase RES concentration to 5 µM, it leads to a significant reduction in cell viability. On the same period of time, a concentration of RES above 10 µM leads to an increase in LDH release (Figure 1b).

Regarding hGC, we found that RES also had an effect on cell variability at different concentrations and times when compared to the control. However, in contrast to COV434, no clear pattern to these findings was observed. In hGC, RES promoted a significant increase in cell viability (Figure 1c). In addition, when we increased RES concentration to 10 µM, even at 72 h, there was no reduction in cell viability. Accordingly, the quantification of LDH released to the medium showed no differences between the control and RES treatments (Figure 1d).

**Figure 2:**
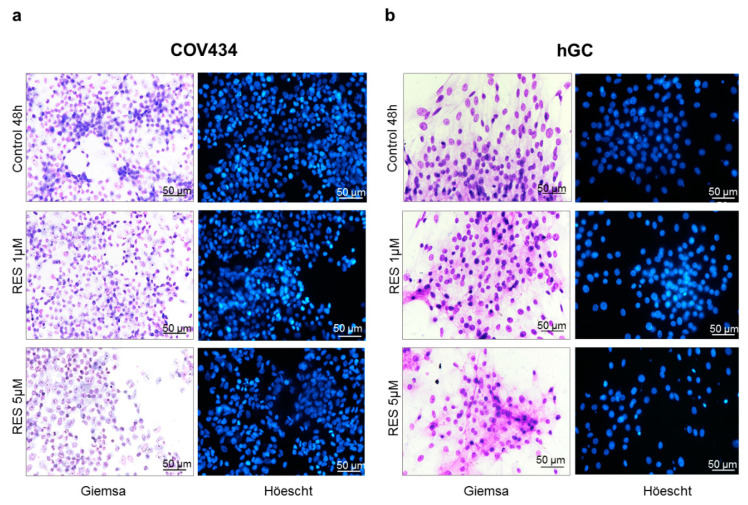
RES effect on COV434 (**a**) and hGC (**b**) morphology. Cell morphology and nuclei were analyzed after 48 h of treatment using Giemsa and Höechst staining, respectively, in the absence (control) and presence of RES (1–5 µM). Results are shown from single representative of three independent experiments. Total magnification 200×.

The concentrations of 1–5 µM of RES were chosen for morphological studies (Figure 2) since COV434 demonstrated a decrease in cell viability at 5 µM. Likewise, we tested the same concentrations during our experiments in primary GC, in order to approximate to physiological concentrations of RES and to be able to compare both cell models. No morphological alterations were observed in both GC models. In addition, no nuclear condensation or fragmentation was noted in Höechst staining.

**Figure 3:**
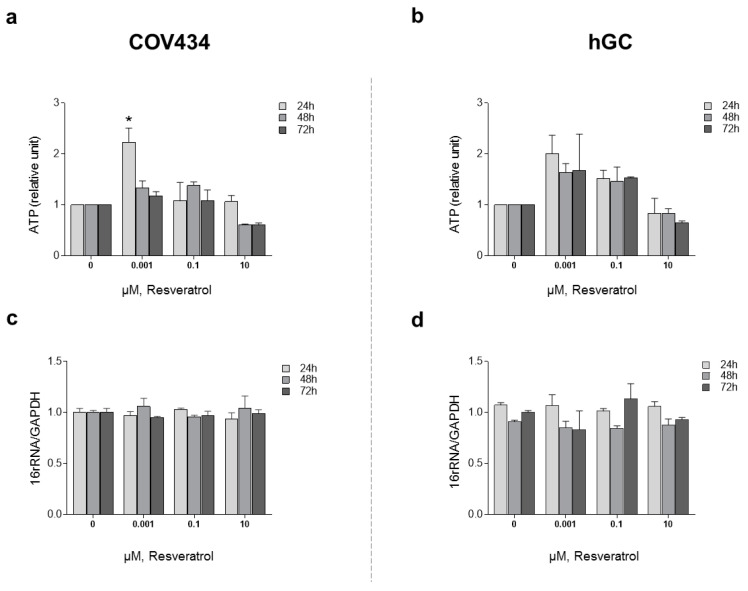
RES effect on mitochondrial function: ATP production. COV434 (**a**) and hGC (**b**) measurement of ATP levels after RES treatment (0.001–10 µM) by a luminescent assay (24, 48, and 72 h). Relative mtDNA copy number was determined using quantitative real-time PCR with primers for the 16S rRNA gene (mitochondrial) and the GAPDH gene (nuclear) for COV434 (**c**) and hGC (**d**). Results were related to the control and expressed as a mean ± SEM of at least three independent experiments performed in triplicate. Significant differences between control and treated cells are indicated as * (*p* ˂ 0.05).

In order to understand the impact of RES treatment on mitochondrial function and mitochondrial membrane potential, we evaluated ATP synthesis and ΔΨ_m_ (Figure 3). To study ATP synthesis, we chose low concentrations of RES (0.001–0.1 µM), which had previously been indicated to increase cell viability, and compared these to a higher concentration (10 µM). Treatments with RES 0.001 µM at 24 h induced a significant increase on ATP production by COV434 mitochondria (Figure 3a). At the same concentration, RES did not induce an increase on ATP production by hGC mitochondria at 24, 48, or 72 h (Figure 3b).

**Figure 4:**
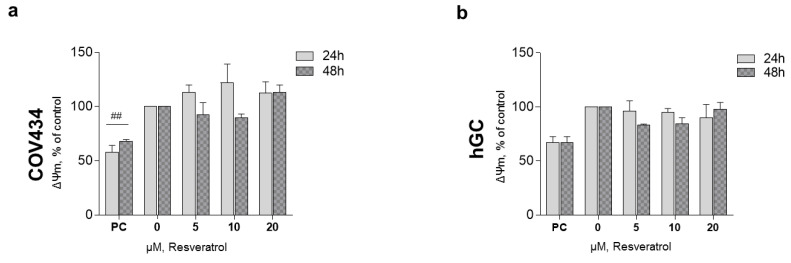
RES effect on granulosa cell (GC) mitochondrial membrane potential. ΔΨ_m_ of COV434 (**a**) and hGC (**b**) after 48 h of treatment with RES (5–20 µM) compared with non-treated cells (control), assessed by fluorescence assay with DiOC_6_ probe. Carbonyl cyanide m-chlorophenylhydrazone (CCCP) (10 µM) was used as a positive control (PC). Results are related to the control and expressed as mean ± SEM of at least three independent experiments performed in triplicate. Significant differences between control and PC are indicated as ^##^ (*p* ˂ 0.01).

After 48 and 72 h, at a RES concentration of 10 µM, ATP synthesis by both cell models demonstrated a tendency to decrease (Figure 3a,b). These observations were not accompanied by alterations in the mitochondrial number as it was shown by the 16RNA/GAPDH ratio in Figure 3c,d. For the highest concentrations, ΔΨ_m_ was measured on COV434 and hGC (Figure 4). Concentrations of 5, 10, and 20 µM were chosen as they result in a significant decrease in COV434 viability. Treatments with RES did not induce significant differences in ΔΨ_m_.

### 3.2. RES Protects GC from Oxidative Stress

**Figure 5:**
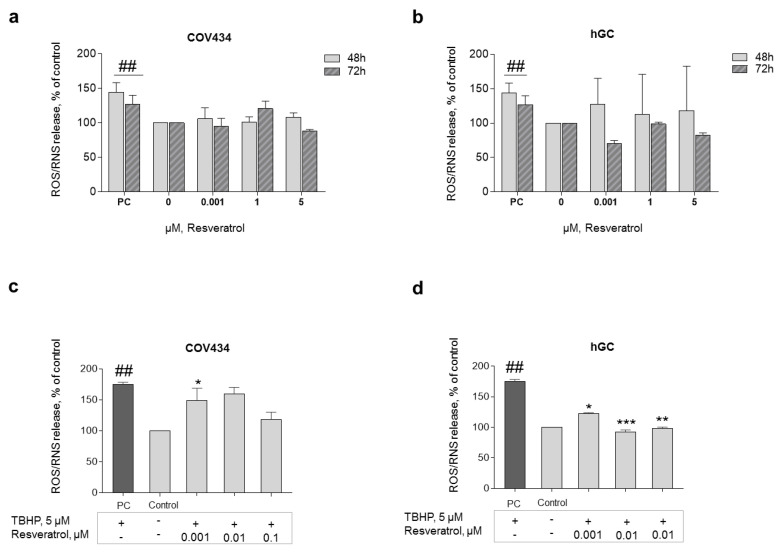
ROS formation after RES addition. The antioxidant capacity of RES was analyzed through the evaluation of ROS production after 48–72 h of treatment with RES (0.001–5 µM) on COV434 (**a**) and primary hGC (**b**). H_2_O_2_ (200 μM) was used as a positive control (PC). Significant differences between PC and cells treated only with medium (control) are denoted as # (*p* ˂ 0.05); ROS production on COV434 (**c**) cells and primary hGC (**d**) treated with RES at different concentrations (0.001–0.1 μM) for 72 h and then co-treated with 5 μM of TBHP (using dimethyl sulfoxide (DMSO) as a solvent), assessed by fluorescence assay with DCFH-DA probe. Significant differences between cells treated with TBHP and control (only cell medium) are marked as ^##^ (*p* ˂ 0.01). Significant differences between cells treated with TBHP alone and RES treated cells are denoted as * (*p* ˂ 0.05), ** (*p* ˂ 0.01), and *** (*p* ˂ 0.001). Results are expressed as mean ± SEM of three independent experiments performed in triplicate.

In order to explore the antioxidant capacity of this natural compound, ROS production was analyzed. To understand the impact of low doses of RES on ROS/RNS production, RES concentrations of 0.001–5 μM were chosen. The results showed no significant ROS/RNS production after RES addition (0.001–5 μM) at 48 or 72 h (Figure 5a,b). Nevertheless, GC primed with RES for 72 h and then co-treated with 5 μM of TBHP (stress inducer) showed a significant drop in ROS production when compared with only TBHP. In this line, statistically significant results were observed after treatment with RES 0.001 μM on COV434 and RES 0.001–0.01 μM on hGC (Figure 5c,d).

### 3.3. RES Impacts GC Estradiol Production

**Figure 6:**
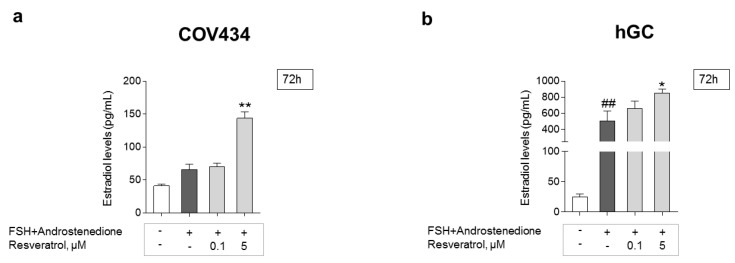
RES effect on the secretion of estradiol by GC. Impact of RES on estradiol secretion after 72 h on COV434 (**a**) and primary hGC (**b**); significant differences between cells treated with follicle-stimulating hormone (FSH) and 4-androstenedione and control are marked as ^##^ (*p* ˂ 0.01). Significant differences between cells treated with different concentrations of RES and cells with only FSH and 4-androstenedione are marked as * (*p* ˂ 0.05), ** (*p* ˂ 0.01). For both cell models, results are expressed as mean ± SEM of at least three (maximum seven) independent experiments performed in triplicate.

GC estradiol secretion was evaluated by the ELFA technique. To understand the possible impact on estradiol production after long periods of RES intake, a 72-h treatment and two different RES concentrations, one lower and one higher, were chosen (Figure 6a,b). Cells treated with RES presented a dose-dependent increase, being significant at 5 μM on both cell models. The addition of FSH + androstenedione resulted in a significant increase in estradiol levels on hGC when compared to the control. This effect was not observed in the COV434 cell line.

## 4. Discussions

Resveratrol is often used in nutritional supplements and it is known as a potent antioxidant and anti-inflammatory compound [33]. Our data showed that RES in high concentrations induced a reduction on cell viability of COV434 cell line. On the other hand, primary GC showed improved viability after treatment with RES. This may be due to the fact that COV434 is a tumoral cell line and the primary GC considered healthy cells. Lang et al. found that RES does not trigger apoptotic mechanisms in healthy ovarian surface epithelial cells. In contrast, other studies have shown that RES induces significant ROS production and triggers oxidative stress in cancer cells [34,35], leading to apoptosis and autophagy in human ovarian cancer [36]. This evidence supports our findings between the two cell models. Other antioxidants such as luteolin [35] and α-lipoic acid [37] have also been related to ROS-induced cell death in cancer cells.

Nevertheless, in our studies, we failed to notice COV434 apoptotic cell death as no suggestive nuclei alterations were observed; nor were target differences in ROS production and mitochondrial membrane potential analysis. Interestingly, Ortega et al. showed that RES in rat ovarian GC induced a biphasic effect on DNA synthesis, inhibiting it at higher concentrations (10–30 μM) [38]. However, morphological studies also reached the same conclusion, as no morphological changes were noted and there were no characteristic evidences of apoptosis such as cell shrinkage or chromatin condensation at concentrations of RES between 10 and 50 μM in GC of mouse ovary [38]. These findings are further corroborated by Morita et al. [39].

Since mitochondria are important for energy production and steroidogenesis [40], in addition to mitochondrial membrane potential, we also assayed the damage on mitochondria function of GC. ATP synthesis by mitochondria was evaluated on both cell models. The presented results suggest that the increase on GC viability (assessed by MTT), with lower concentrations of RES, may be related with ATP production by mitochondria. Although we did not observe significant ATP production at low doses of RES on primary GC, it indicates the potential benefits that this compound may exert on cell viability. Our findings are supported by Ragonese et al., who reported ATP increase after the addition of low doses of RES (3 μM) on human GC [41]. Li et al. showed that 10 μM of RES increased ATP levels in human GC compared with the control group, after 24 h of treatment [42]. Studies regarding mitochondrial functions on different cell models, such as rat liver and bovine heart, described RES as a disruptor of energy metabolism, relating to the suppression of cellular respiration due to membrane damage [43]. Nevertheless, we did not observe a decrease in ATP intracellular levels nor a decrease in the mitochondrial number and potential with RES at 10 μM. Together, this indicates that at low doses, RES did not interfere with mitochondrial activity.

Although RES does not induce ROS/RNS generation after 72 h, this natural compound protects both GC models from stress induction. Accordingly, a study conducted by Kolesarova and collaborators, using porcine GC, suggests that toxicity induced by deoxynivalenol is inhibited by RES, proposing a protective effect by this natural compound [44]. In addition, a recent study suggests that RES may be a potential drug to improve fertility preservation for patients undergoing chemotherapy. The authors demonstrate that RES increases sirtuin 1 expression, as well as decreases oxidative stress and reduces Beclin1, LC3B, Bax, and Caspase-3 levels [45]. Another study on cat’s oocytes demonstrated that RES treatment (5 μM) significantly reduced the level of ROS on oocytes recovered from the ovaries that were stored from 48 h (associated with a progressive increase of ROS) [46].

Regarding steroidogenic function, previous reports of RES reveal that it has a similar affinity with estrogen receptors ERα or ERβ and interferes with the functions of E2 [47]. It has been demonstrated that RES acts as an estrogen agonist, in the absence of 17beta-estradiol, and an antagonist in the presence of 17beta-estradiol [47,48]. Our results indicate that there were no changes in estradiol levels at lower concentrations of RES. However, there was an increase in estradiol production at 5 μM. This observation contrasted with previous observations in rat ovarian GC, where authors demonstrated a decrease in aromatase expression and estrogen production at high concentrations of RES (10–30 μM) [38]. In contrast, Morita and collaborators showed an increase in Steroidogenic Acute Regulatory Protein (StAR) and aromatase levels and an increase in progesterone secretion, using RES (100 μM) in the same cell type [39]. However, using the swine granulosa cell model, the authors demonstrated that RES analogues promote steroidogenesis [49].

Finally, RES supplementation may be of interest for women who have fertility complications, since lower doses of this natural antioxidant represent a decrease on oxidative stress that is highly associated with several reproductive pathologies [50,51]. Based on our results, lower concentration of RES tended to reduce ROS/RNS formation after stress inducement, particularly in primary GC. In addition, hGC viability increased up to 10 μM and estradiol secretion at 5 μM on both cell lines. However, more quality investigation in this area allied to clear evidence is needed, as it is pointed out by a COCHRANE meta-analysis on the impact of antioxidants on female infertility [52]. Interestingly, a novel approach on mitochondria targeting has been explored in recent years, based on molecules that disrupt its function. Several pathways that lead to caspase activation, as well as to morphological and biochemical alterations in the cell, were observed [53]. Due to its function as a double-edged sword, mitochondrial-targeting research has been using RES to directly modulate the oxidative cell environment in different pathologies such as Alzheimer’s disease and within oncotherapies [54,55,56]. In this line, there is still a lack of studies regarding the role of RES in women with poor ART outcomes [54]. This study contributes to understanding the direct effects that may surge within fertility therapies.

## 5. Conclusion

Our work suggests that low doses of RES may promote follicle quality and reduce oxidative stress in the ovarian microenvironment. Lower concentrations of RES have a protective effect against induced-oxidative stress on COV434 and primary GC. The primary GC were more resistant to this natural compound than COV434. In fact, higher concentrations of RES resulted in decreased viability of COV434. Although some evidence points to the beneficial effects of RES on different follicle models [57,58], more quality studies regarding RES dual effects in a human fertility context are needed.
